# Supplementary material for: Ultra-fast single-crystal polymerization of large-sized covalent organic frameworks
Source: Nat Commun. 2021 Aug 23;12:5077. doi: 10.1038/s41467-021-24842-x (PMC8382702; doi:10.1038/s41467-021-24842-x)
Supplement: Supplementary file 1 — Supplementary Information [file 41467_2021_24842_MOESM1_ESM.pdf]

Supplementary Information for

Ultra-Fast Single-Crystal Polymerization of Large-Sized Covalent Organic Frameworks

Peng et al.

Supplementary Note 1. Detailed discussion of angle-dependent photoluminescence.

The polarized photoluminescence (PL) images (Fig. 5a-c) reveal the angle-dependent emission from the sc-COF_{TP-Py} crystal. When the polarization angle (θ) changes from 0 °to 180 °(the long axis direction of the crystal is set to 0 °), the PL emission from the crystal gradually becomes stronger from 0 ° to 90 °, and then becomes weaker from 90 °to 180 °.

The PL spectra of different θ show that the minimal PL intensity (I_{PL}) is detected at θ = 0 °, and as θ approaches 90 °, the I_{PL} reaches the maximum. The dichroic ratio (DR) (which is defined as I_{PL} maximum/I_{PL} minimum) is as high as 43.5, and the polarization ratio (ρ) (which is defined by $\rho = (DR - 1)/(DR + 1)$) is 0.96. To the best of our knowledge, this is the first report of polarized PL emission from COF materials, and this result (ρ) is among the highest values for organic or polymeric materials[1]. As a comparison, the ρ of lanthanide metal-organic framework single crystals[2] is 0.84, the ρ of the thiophene-based oligomer single crystals[3] is 0.87, the ρ of organic crystals based on ionic perylenemonoimide[4] is 0.80, the ρ of single crystalline nanobelts of perylene-carboxylic diimides[5] is 0.82, the ρ of organic microbelts generated by hydrogen bonding between guanidinium cations and stilbene-based sulfonate anions[6] is 0.71. This phenomenon only occurs in the case of sc-COF_{TP-Py}. No angle-dependent emission can be observed in the case of os-COF_{TP-Py} (Fig. 5 a, b).

The PL properties of the sc-COF_{TP-Py} crystals are decided by the microscopic arrangement of chromophoric groups and the interactions between each other. The PL emission arises from pyrene groups, and the dipole moment forms an angle of ∼90 °with the long axis of the sc-COF_{TP-Py} crystal. Considering the A-A stacking of sc-COF_{TP-Py}, the intra-layer distances (~2.4 nm) of the chromophoric groups are much larger than the inter-layer distances (~0.34 nm). Such an anisotropic arrangement of the chromophoric groups results in angle-dependent PL emission with a ρ up to 0.96. This value also

unambiguously verifies the single crystalline nature as well as the perfect alignment of chromophoric groups within the crystal, indicating the ultra-high crystalline quality of sc-$COF_{TP\text{-}Py}$.

Supplementary Note 2. Detailed discussion of FP-TRMC results.

FP-TRMC is a powerful, electrodeless and reliable technique for determining the conductivities and intrinsic charge-carrier mobilities of conjugated polymers, photo-sensitised materials, discotic liquid crystals, and metal-containing organic compounds[7]. In FP-TRMC, charge carriers are generated upon photo excitation and the local motion of the carriers can be probed via dielectric loss of the low power microwave probes. Thus there is no need to use any electrodes, which minimizes the influence of factors such as interfacial (between the electrode and testing materials) charge injection, interfacial defects, grain boundaries, etc.

In our measurement, after laser pulse exposure under oscillating electric field in a resonant cavity (quality factor: Q ~ 2500), rise and decay profiles of the reflected microwave power (ΔP_r) from the cavity is directly converted into the transient dielectric loss ($\Delta 1/Q$) as Supplementary Equation 1,

$$\Delta P_{\mathrm{r}} = \Delta R P_{\mathrm{r}} \propto \Delta \frac{1}{Q} \tag{1}$$

where the differential reflection coefficient of ΔR is given by Supplementary Equation 2,

$$\Delta R \approx \frac{\Delta \frac{1}{Q}\left(\frac{2}{Q_U} - \frac{2}{Q_C}\right)}{\left(\frac{2\Delta\omega}{\omega_0}\right)^2 + \left(\frac{1}{Q_L}\right)^2} \tag{2}$$

Here the transient change in the resonant frequency by photo-carrier injection ($\Delta\omega$) is negligible, giving a direct estimate of ΔR from static values of Q factors at unloaded (Q_U), loaded (Q_L), coupled with the external microwave circuit (Q_C) and the resonant frequency (ω_0) of the cavity employed. The value of $\Delta 1/Q$ is correlated directly to the electrical conductivity change ($\Delta\sigma$) as Supplementary Equation 3,

$$\Delta\frac{1}{Q} = \frac{\Delta\sigma}{\omega\varepsilon'} \qquad (3)$$

According to Ohm's law, the electrical conductivity change caused by the differential photocarrier injection (Δn) can be given by Supplementary Equation 4,

$$\Delta\sigma = e\Delta n\sum\mu \qquad (4)$$

where e is an elementary charge. The value of Δn is simply represented with the factor (Filling Factor: F_l) of light absorptivity of the testing material matching with the slope of electric field in the cavity as Supplementary Equation 5,

$$\Delta n = I_0 F_l \Phi \qquad (5)$$

Based on the Supplementary Equations of 1, 3, 4 and 5, finally we obtain a following direct expression of form for charge carrier mobility of the carriers by Supplementary Equation 6,

$$\Phi\sum\mu = \frac{1}{eAI_0F_l}\cdot\left(\frac{\Delta P_r}{P_r}\right) \qquad (6)$$

where A can be derived from the above constants as a sensitivity factor (S^{-1} cm).

In FP-TRMC measurement, the observable region of charge-carrier mobility can be extended to a very short time region (end-of-pulse value is on the nanosecond scale) by the high frequency of microwaves. FP-TRMC measurement probes charge carrier motion

within a nanoscale regime. In our measurement, the spatial size of the oscillating motion of charge carriers is estimated within several nanometers at a maximum[8]. Thus, the grain and/or domain boundary will not affect the $\phi\Sigma\mu$ obtained by FP-TRMC. The FP-TRMC result is not related to the grain size but the actual quality (or crystallinity) of the as-grown COFs[9].

The sample quality is of great importance for the practical application of COFs. In this research, the FP-TRMC is performed to evaluate the intrinsic quality of the sc-COFs, which avoids the influence from the crystal size and the sample morphology. After 5 min growth in sc-CO_2, the photo-induced transient conductivity profile of sc-$COF_{TP\text{-}Py}$ has a rapid rise with a maximum $\phi\Sigma\mu$ value of 5.8×10^{-6} cm^2 V^{-1} s^{-1} at a photon density of 1.8×10^{16} photons cm^{-2}, and $\phi\Sigma\mu$ reaches ~6.8×10^{-6} cm^2 V^{-1} s^{-1} after growth in sc-CO_2 for 20 min to 12 hours (Fig. 6). This result indicates that high-quality COFs can be produced within minutes. As a comparison, $\phi\Sigma\mu$ of os-$COF_{TP\text{-}Py}$ samples cannot reach the same value, even after 3 days. Therefore, the supercritically-solvothermal synthesis has remarkable advantage in fast preparation of high quality COFs, compared with the existing preparation methods.

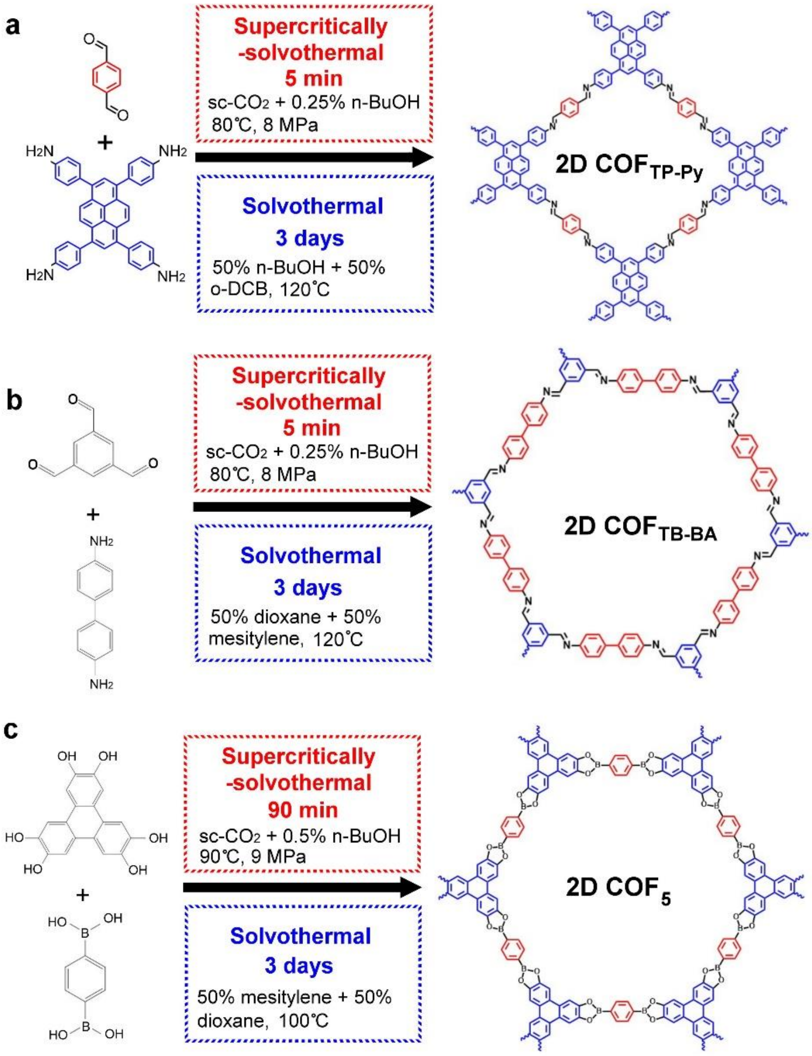

Supplementary Fig. 1. **The polymerization reactions for producing COFs.** Schematic representation of the synthesis of $COF_{TP\text{-}Py}$ (**a**), $COF_{TB\text{-}BA}$ (**b**), COF_5 (**c**) by supercritically-solvothermal or solvothermal polymerization.

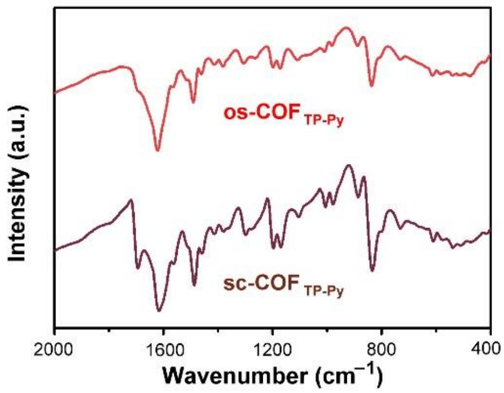

Supplementary Fig. 2. **FT-IR characterization.** FT-IR spectra of sc-$COF_{TP\text{-}Py}$, os-$COF_{TP\text{-}Py}$.

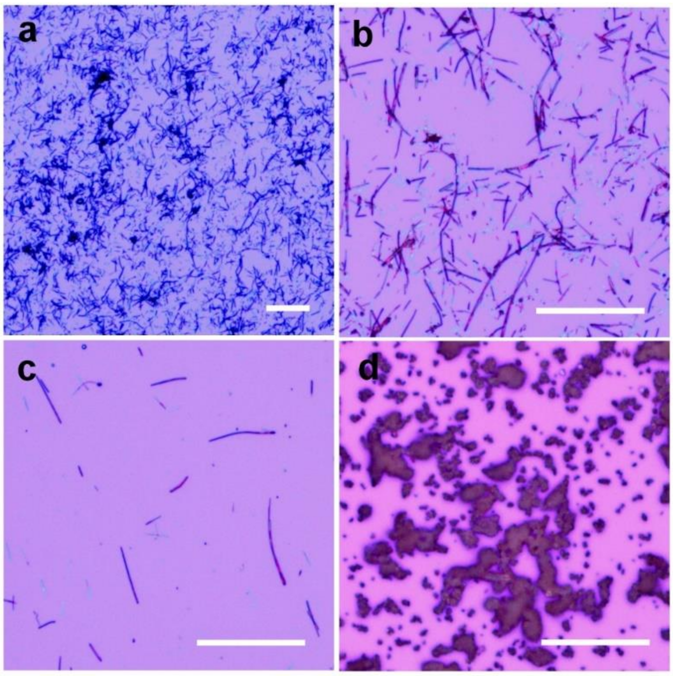

Supplementary Fig. 3. **OM images of the as-grown samples. a-c**, The sc-$COF_{TP\text{-}Py}$ grown for 5 min. **d,** The os-$COF_{TP\text{-}Py}$ grown for 3 days. The scale bars are 50 μm.

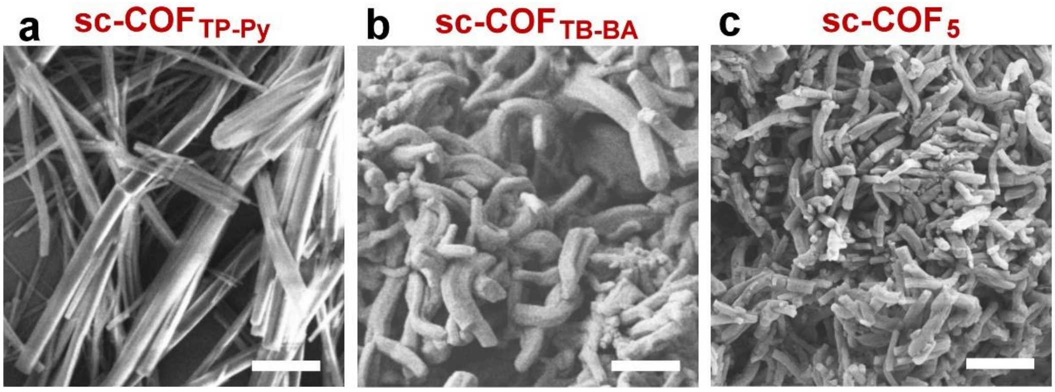

Supplementary Fig. 4. **SEM images of the sc-COFs grown in sc-CO_2. a,** sc-COF_{TP-Py}. **b,** sc-COF_{TB-BA}. **c,** sc-COF_5. The scale bars are 2 μm.

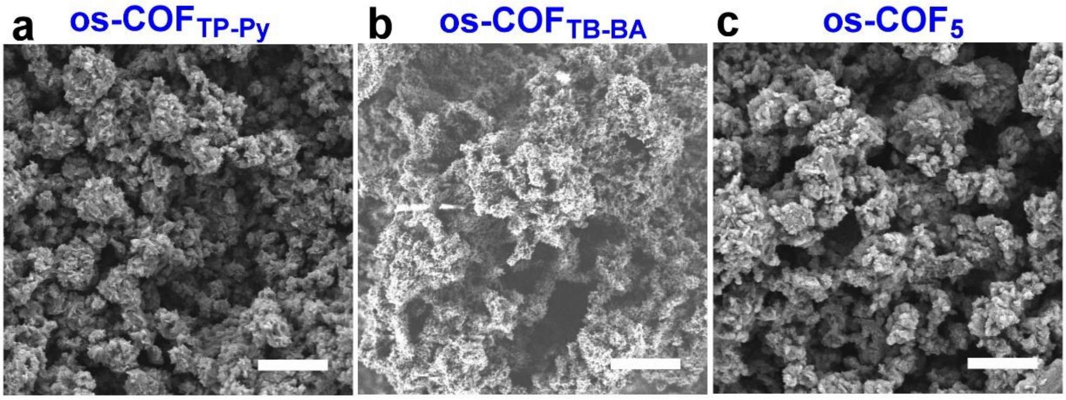

Supplementary Fig. 5. **SEM images of the os-COFs grown for 3 days. a,** os-COF$_{TP\text{-}Py}$. **b,** os-COF$_{TB\text{-}BA}$. **c,** os-COF$_5$. The scale bars are 2 μm.

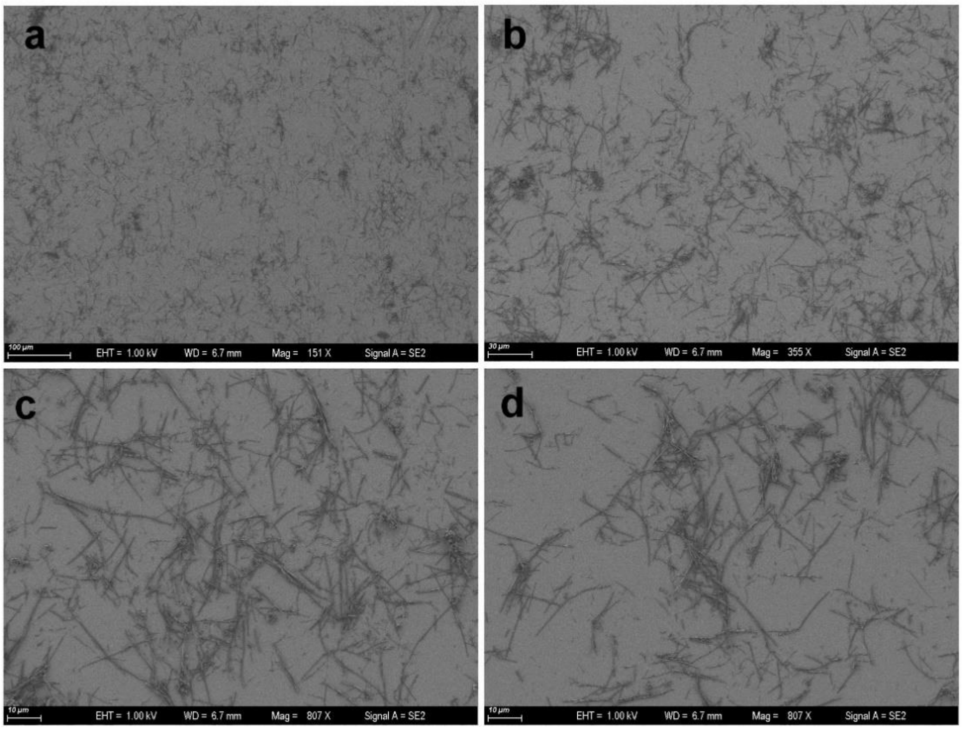

Supplementary Fig. 6. SEM images of the sc-COF$_{\mathbf{TP\text{-}Py}}$ grown for 5 min. The scale bars are 100 μm in **a**, 30 μm in **b**, 10 μm in **c** and **d**.

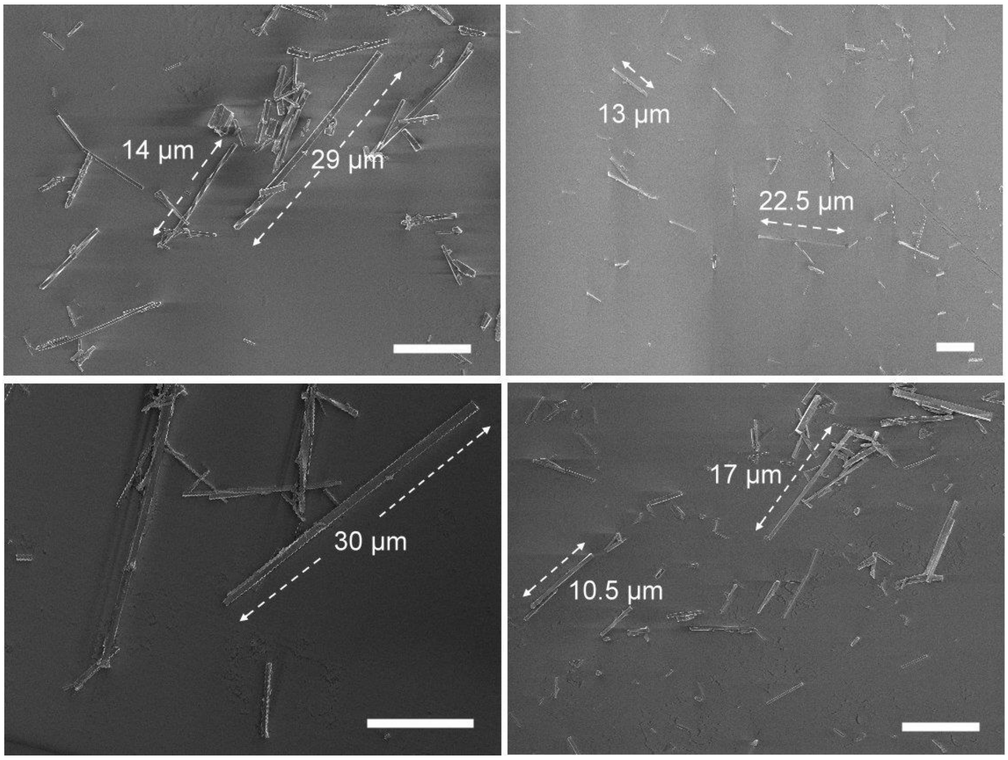

Supplementary Fig. 7. SEM images of sc-COF$_{TP-Py}$ grown for 2 min. The scale bars are 10 μm.

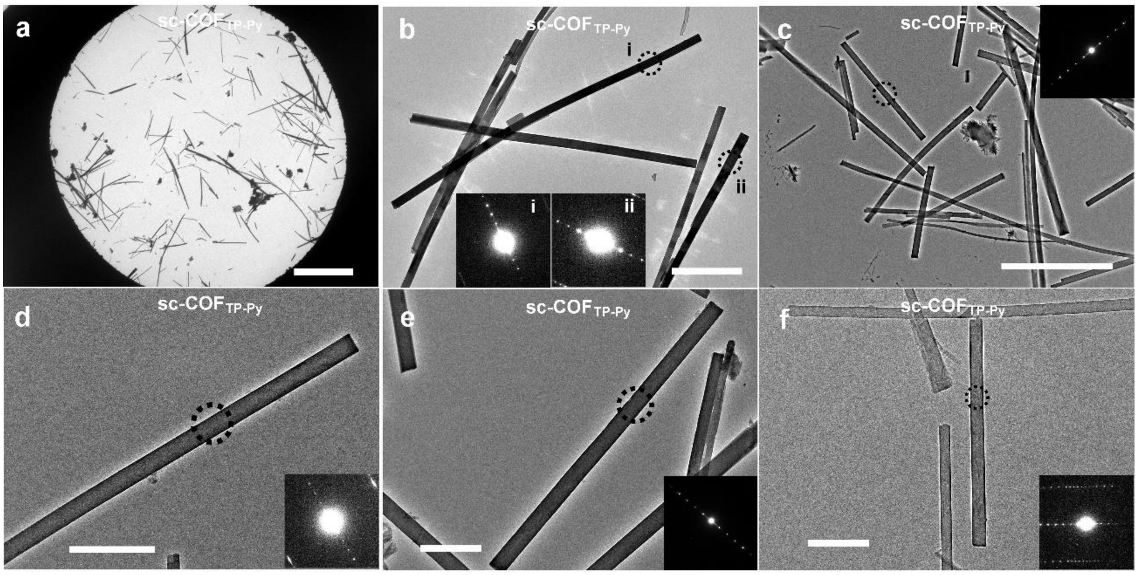

Supplementary Fig. 8. TEM images and SAED patterns of sc-COF$_{TP-Py}$. The scale bars are 20 μm in **a**, 2 μm in **b**, 5 μm in **c**, 1 μm in **d-f**.

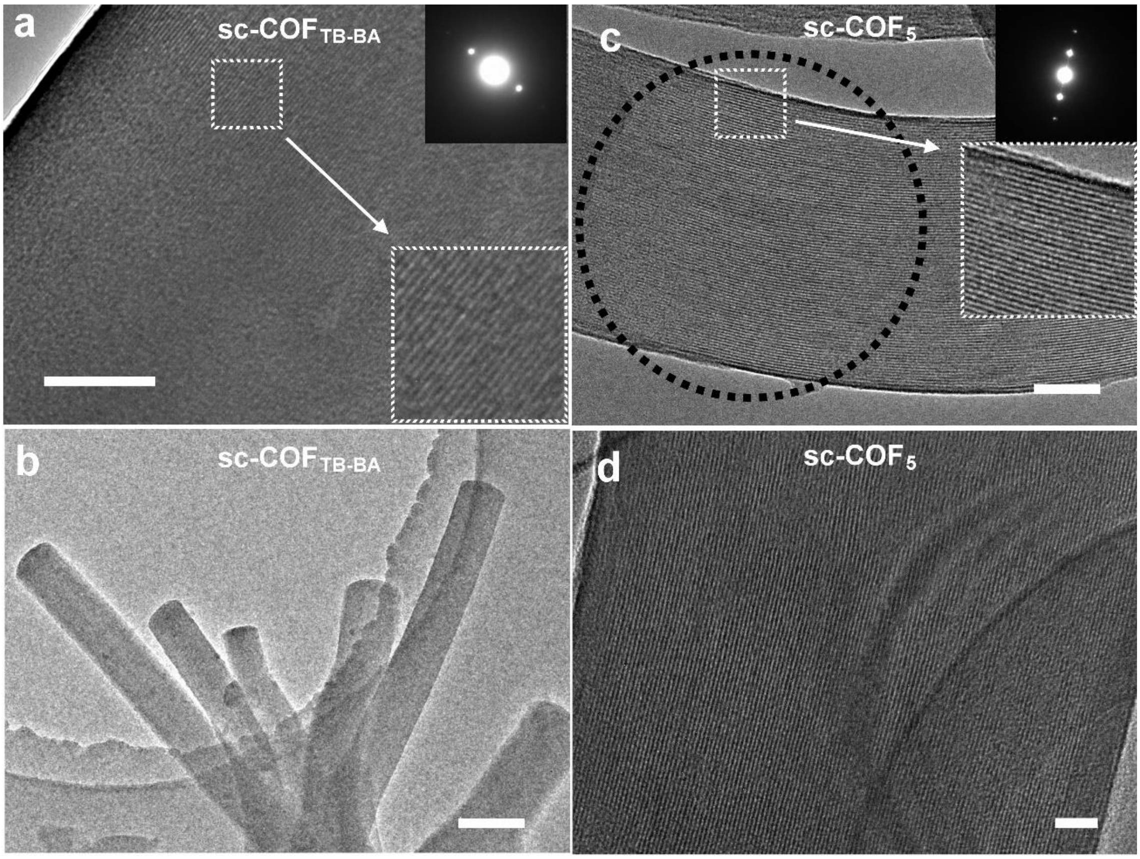

Supplementary Fig. 9. TEM images and SAED patterns of other 2D sc-COFs. a, b, sc-$COF_{TB\text{-}BA}$. **c, d,** sc-COF_5. All of the sc-COFs are highly crystalline with rod like morphologies. The scale bars are 50 nm in **a**, **c**, 200 nm in **b**, 20 nm in **d**.

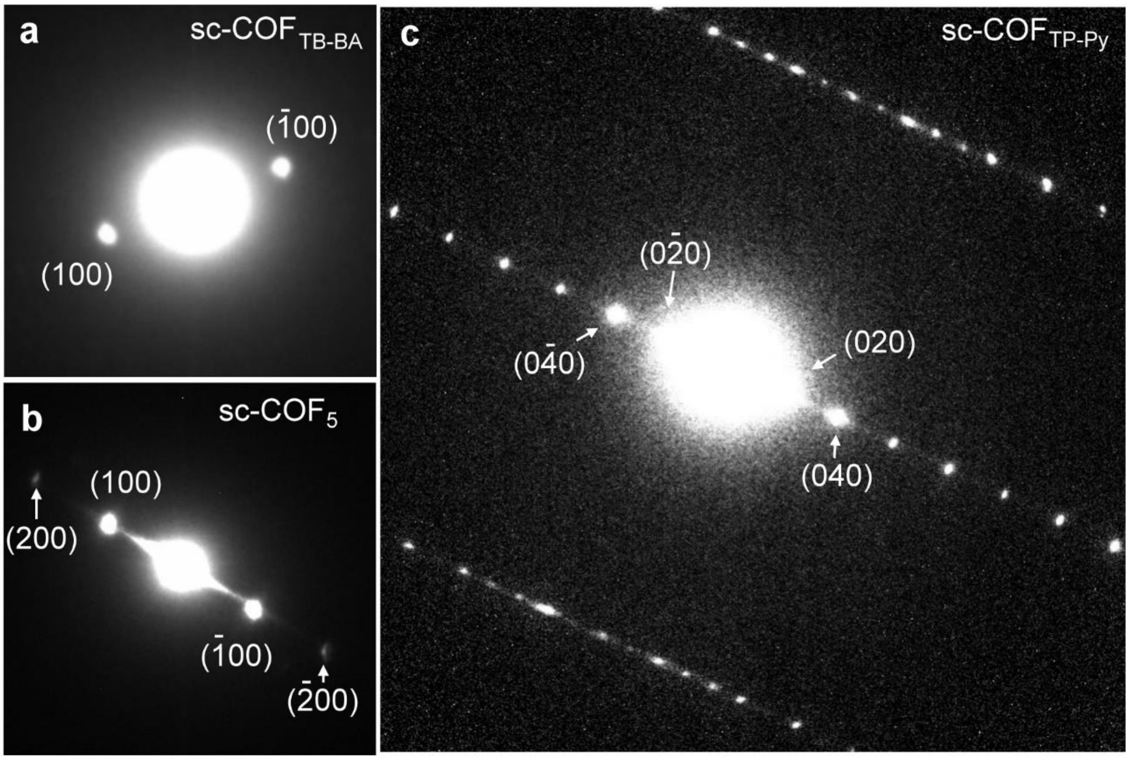

Supplementary Fig. 10. Enlarged SAED patterns of sc-COFs. Sc-COF$_{TB-BA}$ **(a)**, sc-COF$_5$ **(b)**, sc-COF$_{TP-Py}$ **(c)**.

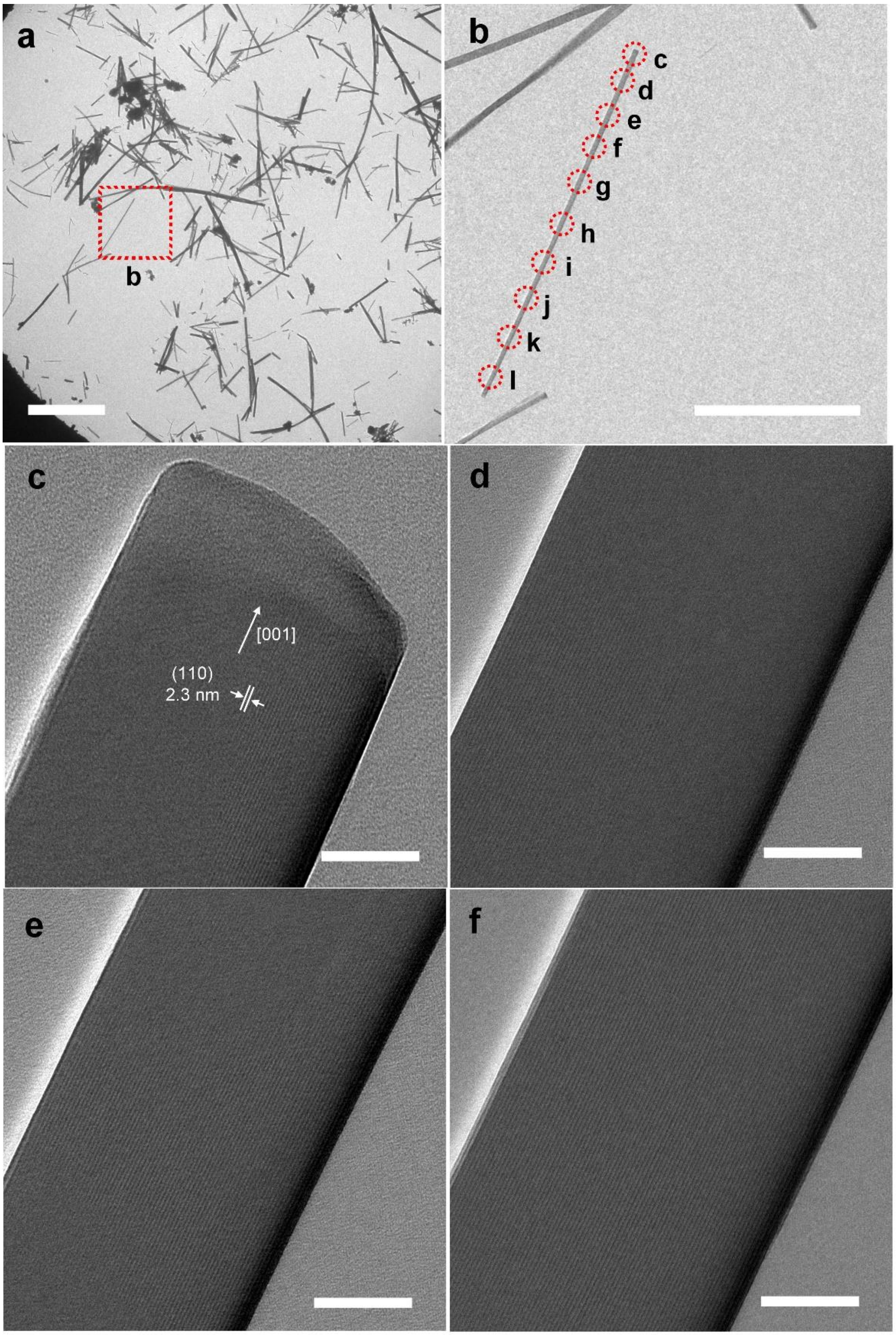
a
b
b
c
d
e
f
g
h
i
j
k
l
c
[001]
(110)
2.3 nm
d
e
f

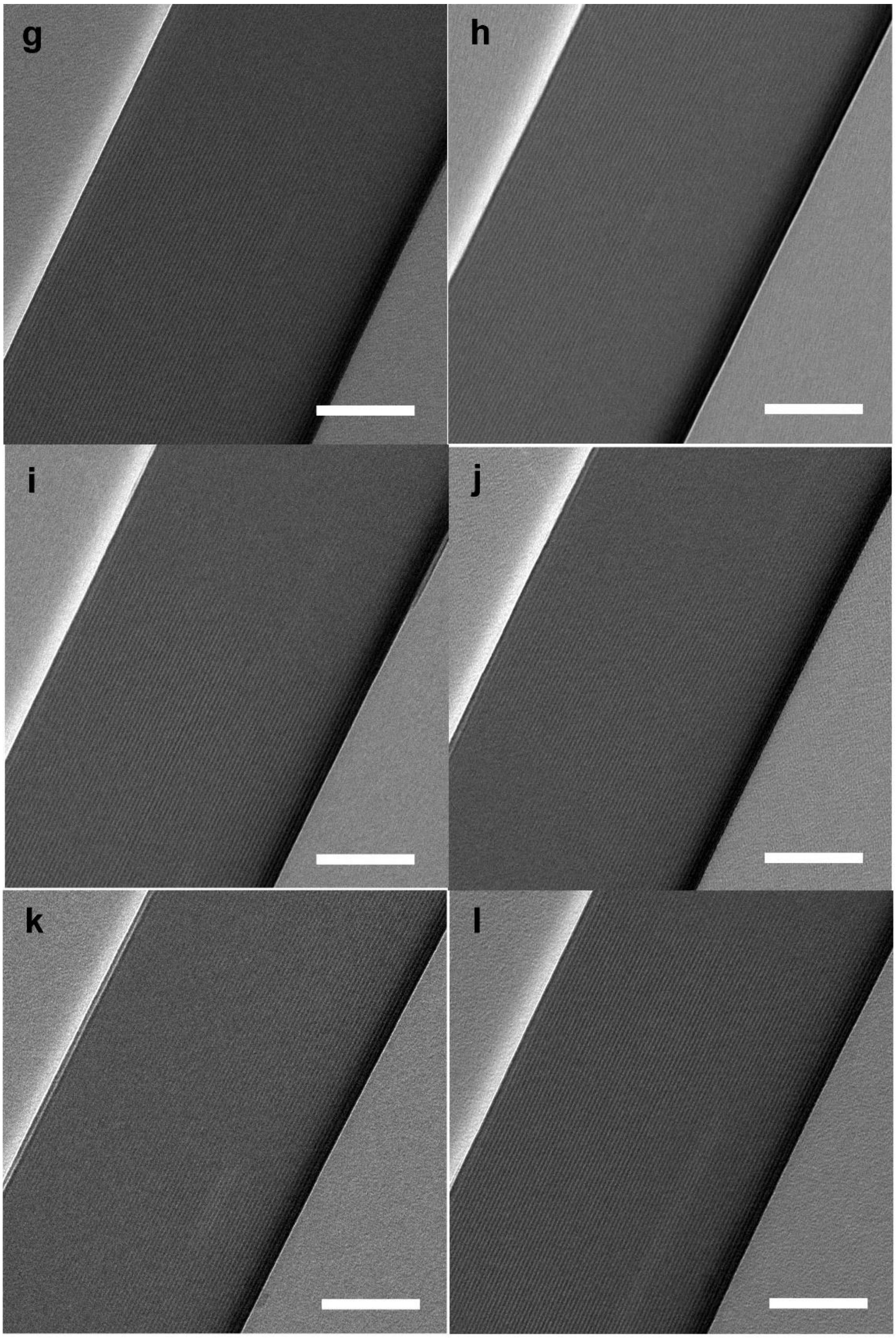

Supplementary Fig. 11. TEM images of a sc-COF$_{TP-Py}$ crystal. a, TEM image and **b,** enlarged image collected from the dashed area in **a. c-l,** Enlarged TEM images collected from the dashed areas in **b**. The scale bars are 10 μm in **a**, 5 μm in **b**, 50 nm in **c-l**.

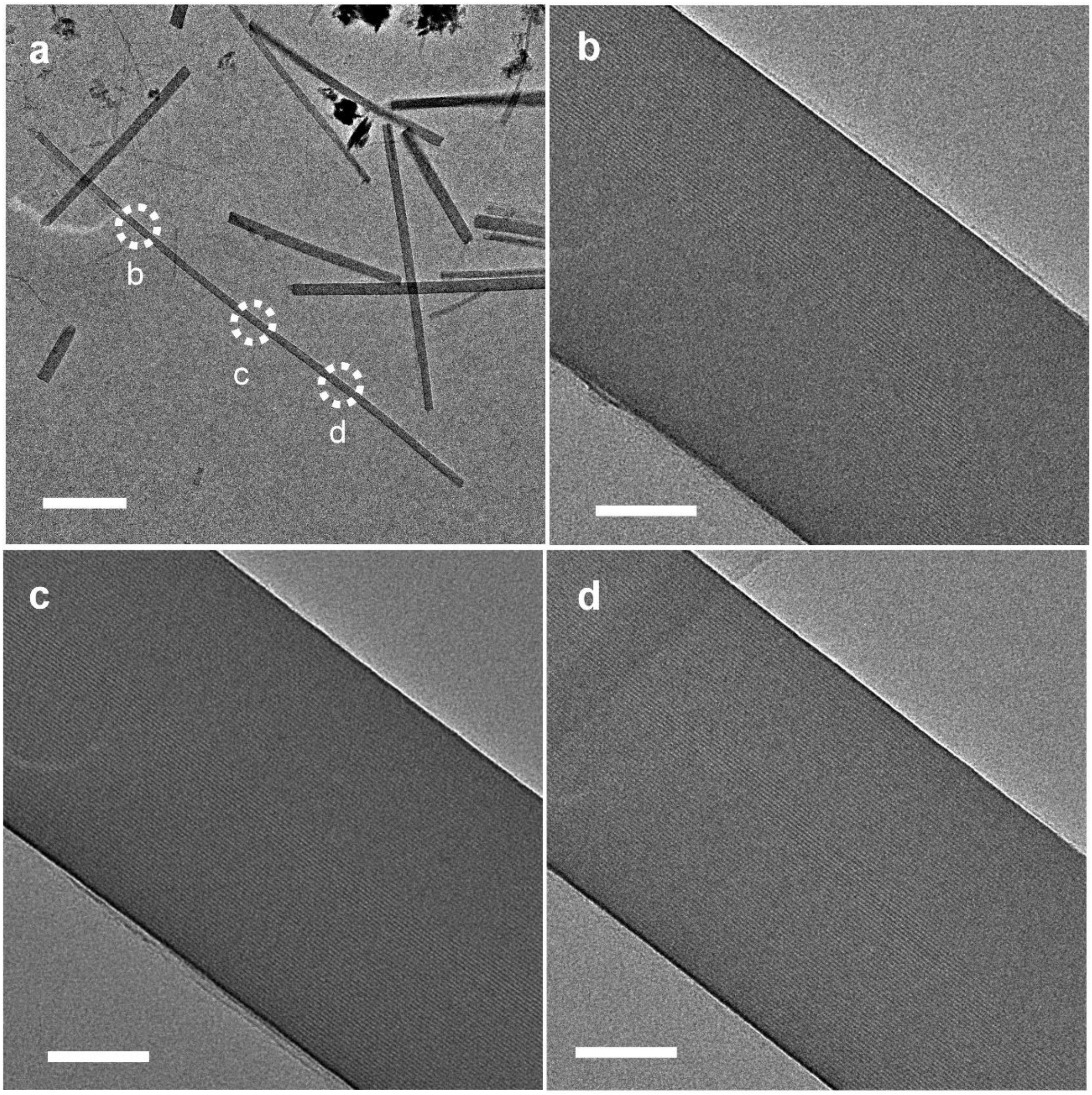
a
b
c
d
b
c
d

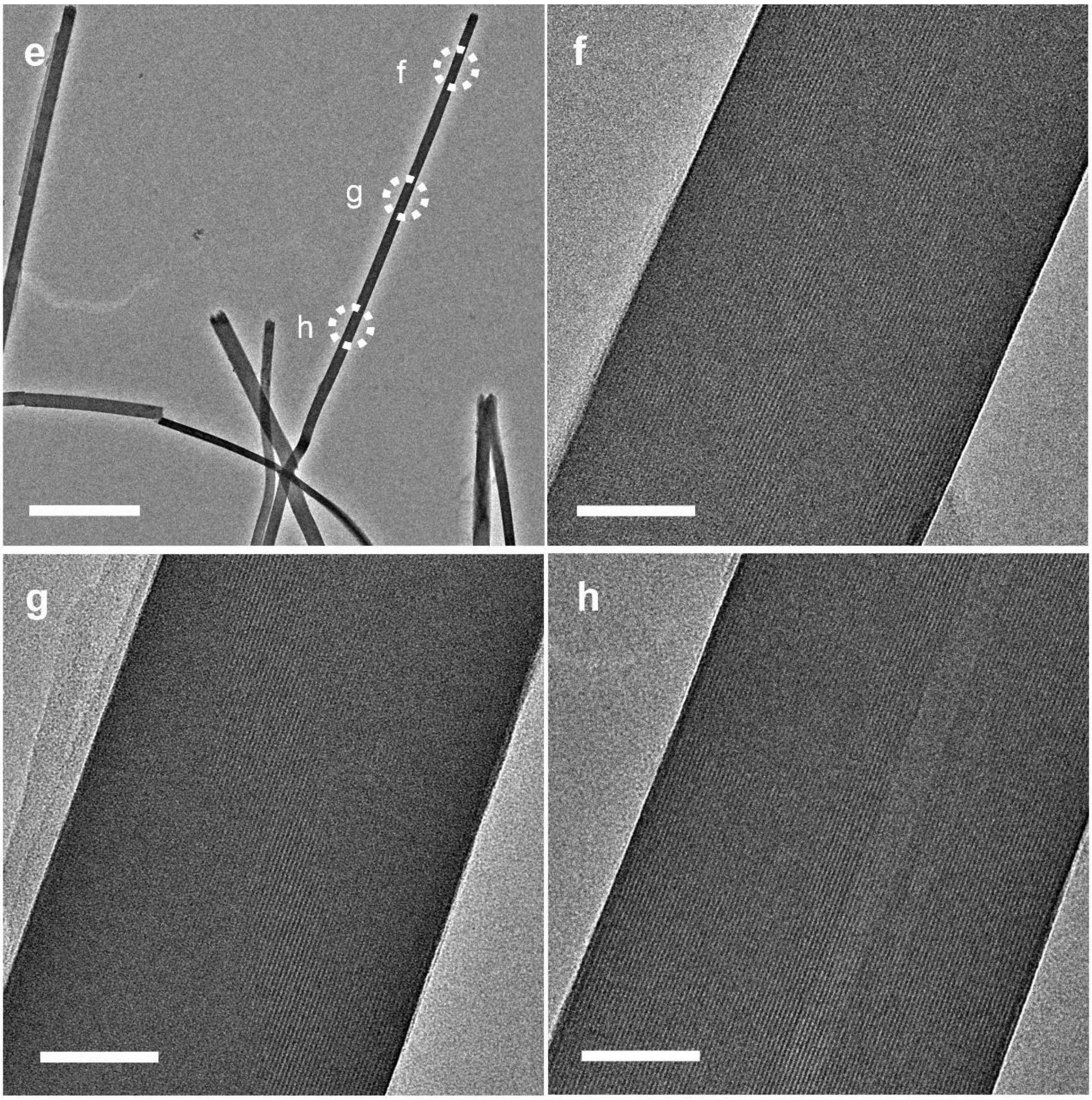

Supplementary Fig. 12. TEM images of two sc-COF$_{\mathbf{TP\text{-}Py}}$ crystals. a, TEM image and **b-d,** enlarged images collected from the dashed areas in **a**. **e,** TEM image and **f-h,** enlarged images collected from the dashed areas in **e**. The scale bars are 2 μm in **a** and **e**, 50 nm in **b-d** and **f-h**.

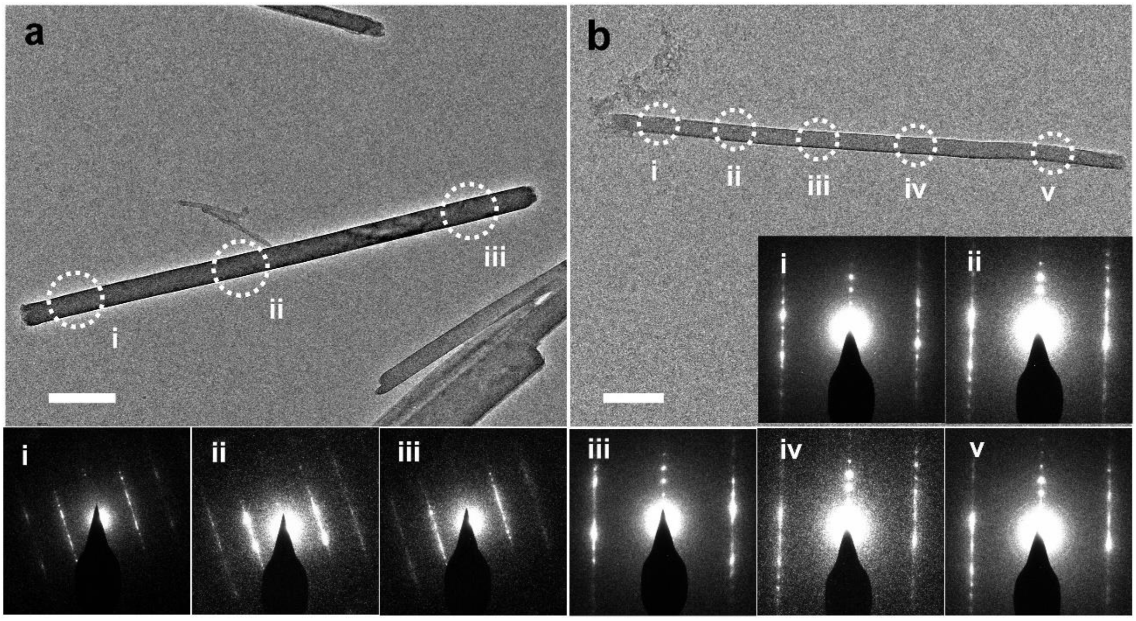

Supplementary Fig. 13. TEM images and SAED patterns of sc-COF$_{TP-Py}$ crystals. The SAED patterns are collected from the regions marked by dashed circles in the TEM images. A same set of SAED patterns are obtained from different locations of the crystal, indicating the single crystalline nature of the sample. The scale bars are 500 nm.

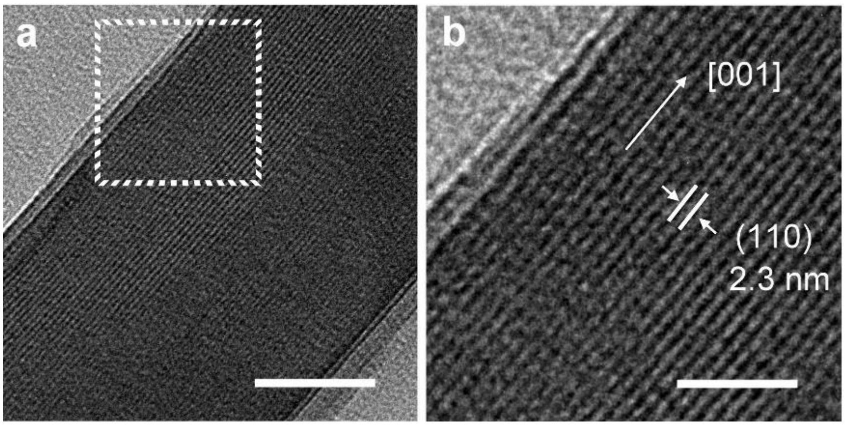

Supplementary Fig. 14. TEM images of sc-COF$_{TP-Py}$ grown for 2 min. b is collected from the dashed area in **a**. The scale bars are 50 nm in **a**, 20 nm in **b**.

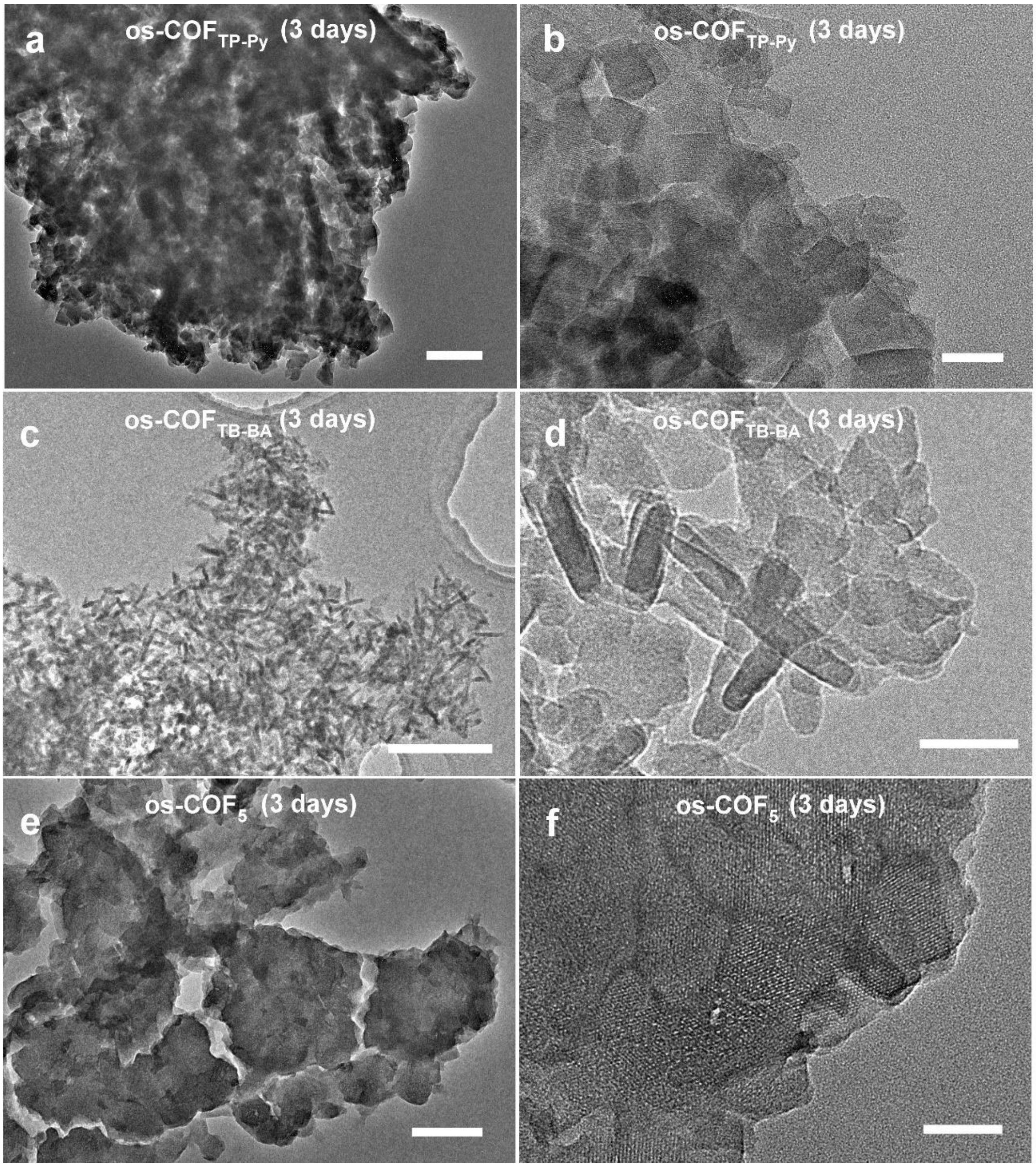

Supplementary Fig. 15. TEM images of os-COFs. a, b, os-COF$_{TP-Py}$. **c, d,** os-COF$_{TB-BA}$. **e, f,** os-COF$_5$. The scale bars are 200 nm in **a**, **e**, 500 nm in **c**, 50 nm in **b**, **d**, **f**.

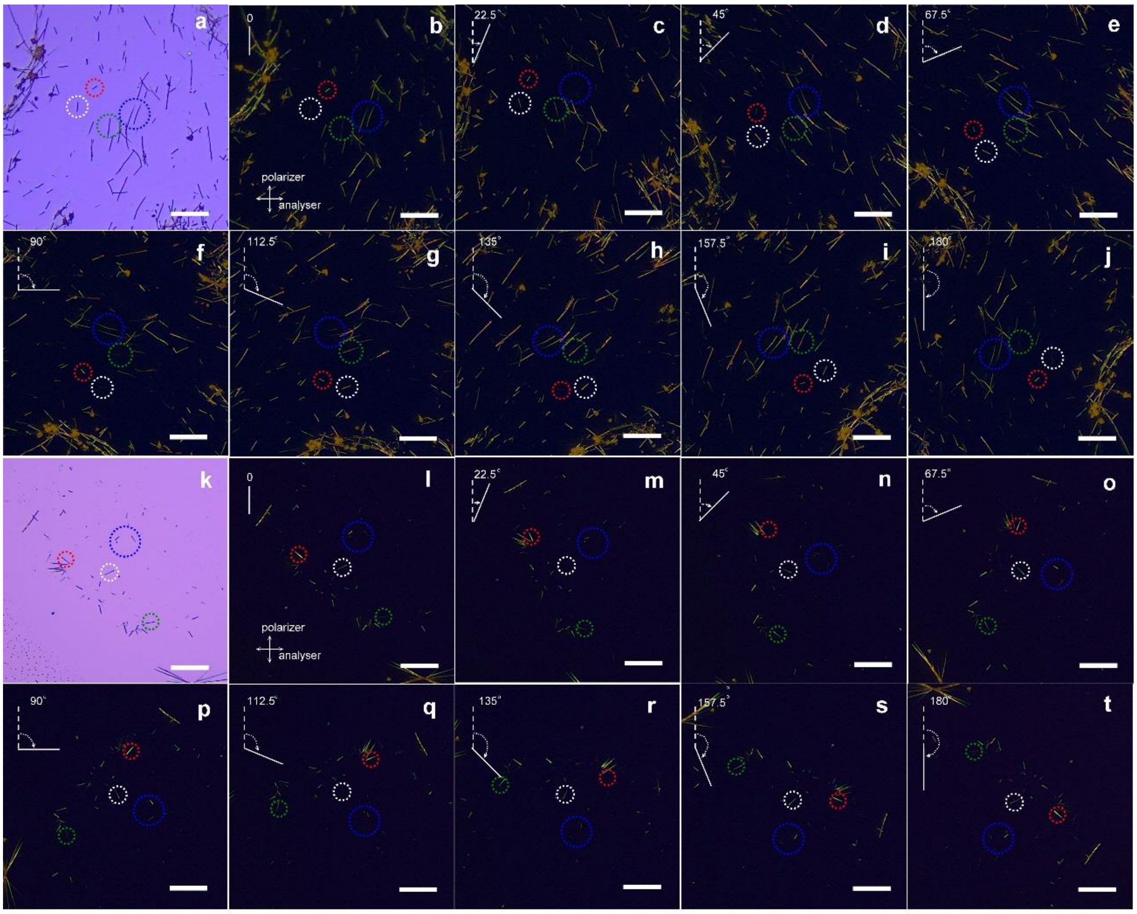

Supplementary Fig. 15. OM and cross-polarized OM images of sc-COF$_{TP-Py}$. a, OM image of sc-COF$_{TP-Py}$ crystals on a Si/SiO$_2$ wafer. **b-j,** Cross-polarized OM images when the wafer is rotated by an angle of 0 °, 22.5 °, 45 °, 67.5 °, 90 °, 112.5 °, 135 °, 157.5 °, 180 °, respectively. **k,** OM image of sc-COF$_{TP-Py}$ crystals on a Si/SiO$_2$ wafer. **l-t,** Cross-polarized OM images when the substrate is rotated by an angle of 0 °, 22.5 °, 45 °, 67.5 °, 90 °, 112.5 °, 135 °, 157.5 °, 180 °, respectively. Uniform polarized light extinction is observed over entire length of the rods, indicating the single crystalline nature of the samples. The scale bars are 50 µm.

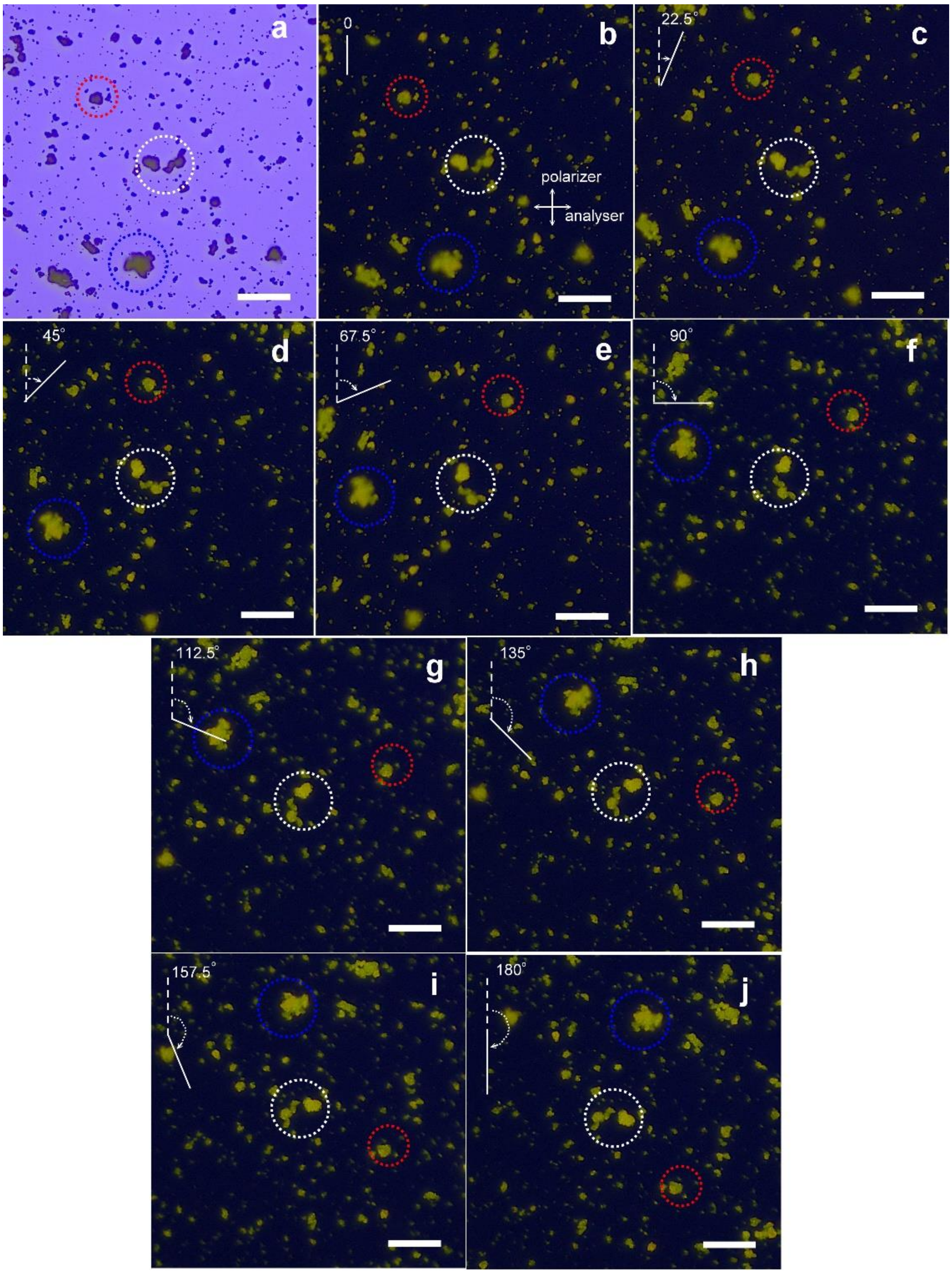

Supplementary Fig. 17. OM and cross-polarized OM images of os-COF$_{TP-Py}$. a, OM image of os-COF$_{TP-Py}$ on a Si/SiO$_2$ wafer. **b-j,** Cross-polarized OM images when the wafer is rotated by an angle of 0 °, 22.5 °, 45 °, 67.5 °, 90 °, 112.5 °, 135 °, 157.5 °, 180 °, respectively. No polarized light extinction is observed, indicating the samples are not single crystals. The scale bars are 50 μm.

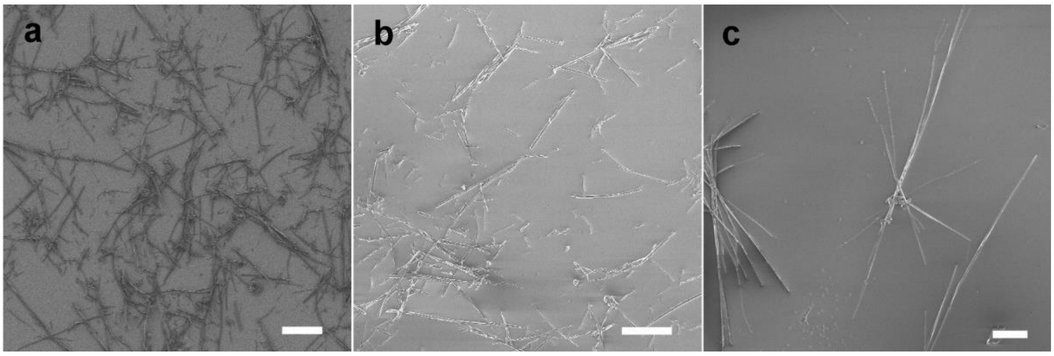

Supplementary Fig. 18. SEM images of sc-COF$_{TP-Py}$ ~~and sc-COF$_{300}$.~~ a-c, sc-COF$_{TP-Py}$ grown for 5 min (**a**), 2 hours (**b**) and 12 hours (**c**). ~~**d-f,** sc-COF$_{300}$ grown for 5 min (**d**), 2 hours (**e**) and 12 hours (**f**).~~ The scale bars are 10 μm.

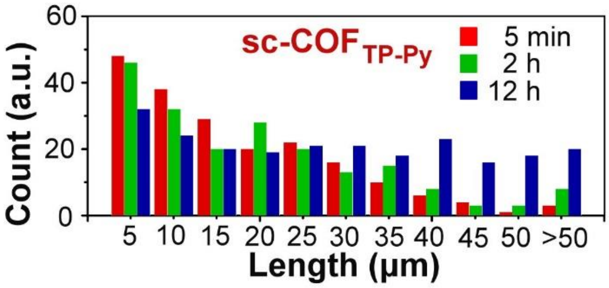

Supplementary Fig. 19. The crystal length grown for different time. Length distribution of the sc-COF_{TP-Py} single crystals grown for 5 min, 2 hours and 12 hours.

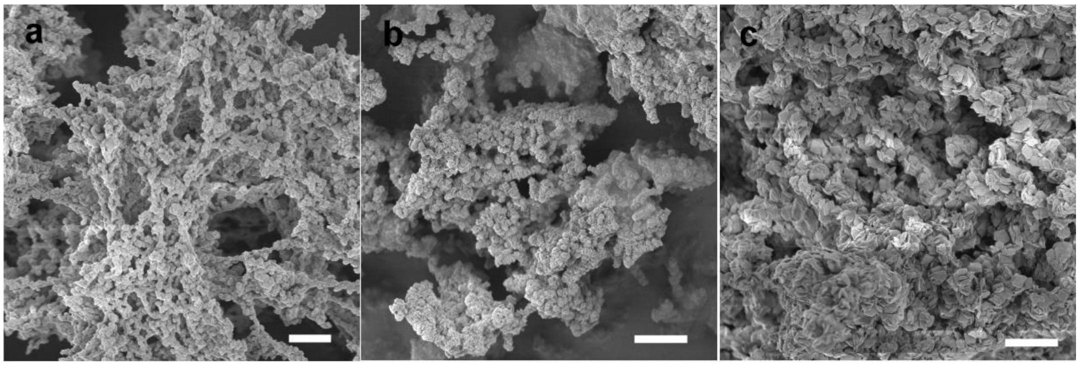

Supplementary Fig. 20. SEM images of os-COF$_{TP-Py}$. a-c, os-COF$_{TP-Py}$ grown for 5 min (**a**), 2 hours (**b**) and 12 hours (**c**). The scale bars are 1 μm.

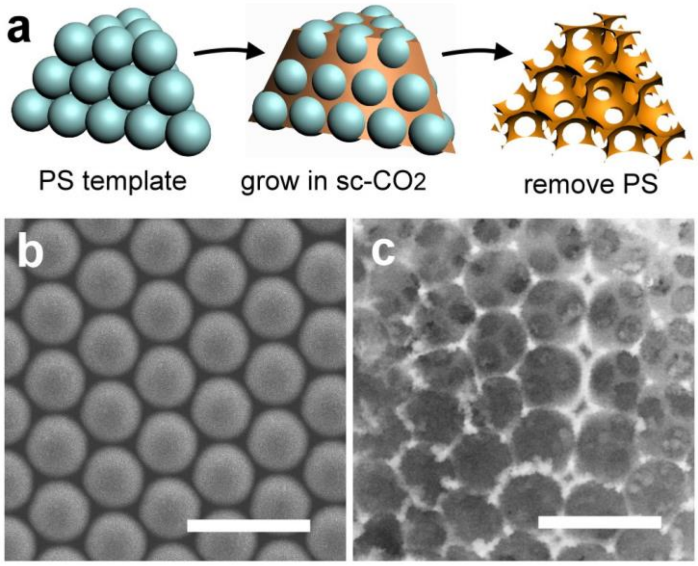

Supplementary Fig. 21. 3D ordered sc-COFs. a, Schematic of the growth of sc-COFs in the small interstices of PS 3D monolith. **b,** SEM image of the PS 3D monolith. **c,** SEM image of the sc-COFs grown in small interstices of PS 3D monolith by supercritically-solvothermal method. The scale bars are 2 μm. This result indicates the high diffusion and penetrating ability in sc-CO_2, which results in the synthesis of COFs in the small interstices.

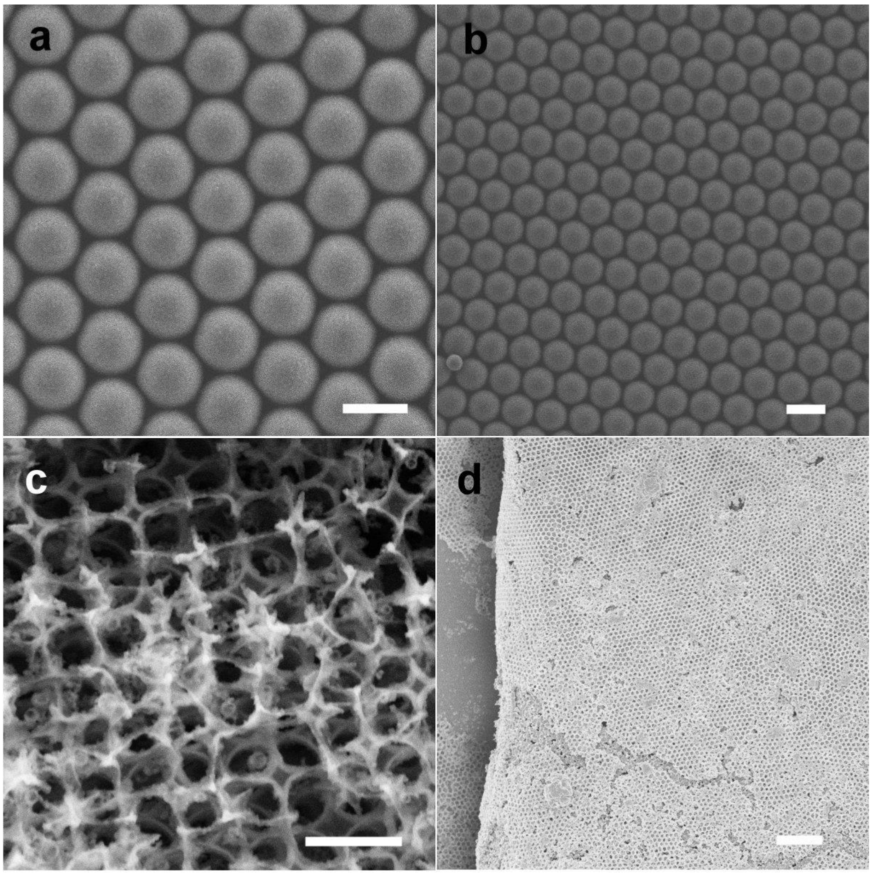

Supplementary Fig. 22. SEM images of 3D ordered sc-COFs. SEM images of **a, b,** the highly ordered 3D monolith of PS and **c, d,** the sc-COFs grown in small interstices by supercritically-solvothermal synthesis. After removing the PS, we observe that the sc-COFs are synthesized in the interstices. The scale bars are 1 μm in **a-c**, 10 μm in **d**.

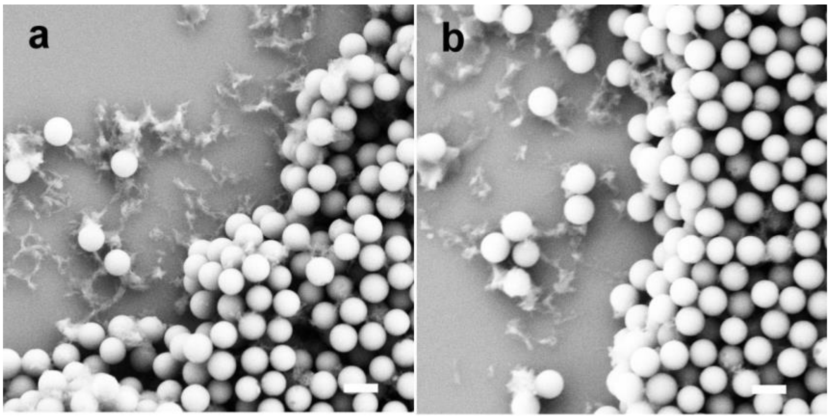

Supplementary Fig. 23. os-COFs grown in 3D monolith of PS. SEM images of the os-COFs in the interstices of the 3D monolith by traditional solvothermal synthesis. It is clear that the os-COFs cannot ben grown in the interstices. The scale bars are 1 μm.

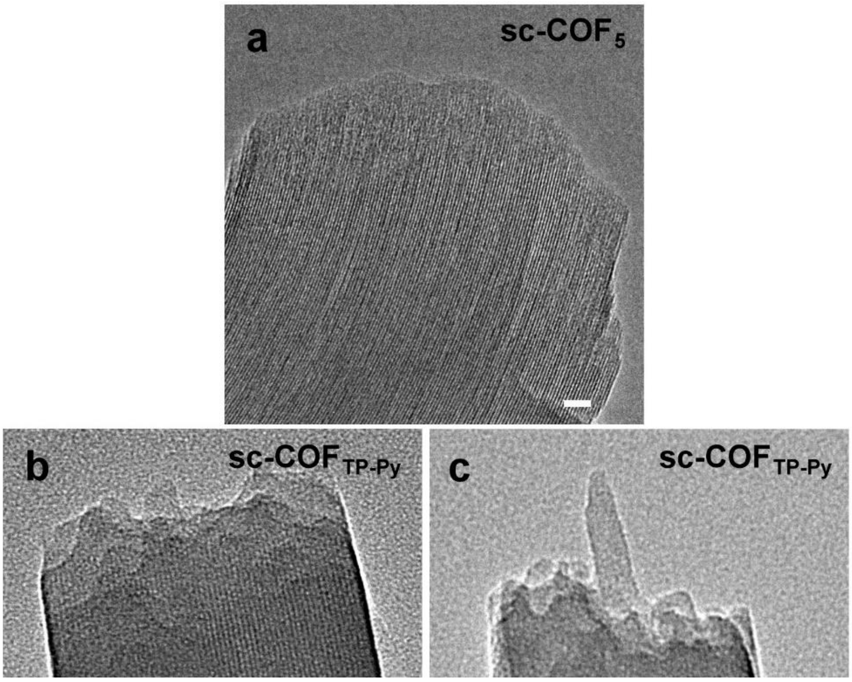

Supplementary Fig. 24. The end of the 2D sc-COF crystals. TEM images of an end of a sc-COF_5 crystal. The crystal nucleus on the end has the same crystalline lattice with the older crystal, indicating the vertically epitaxial growth of the COF crystal from the end. The scale bars are 20 nm.

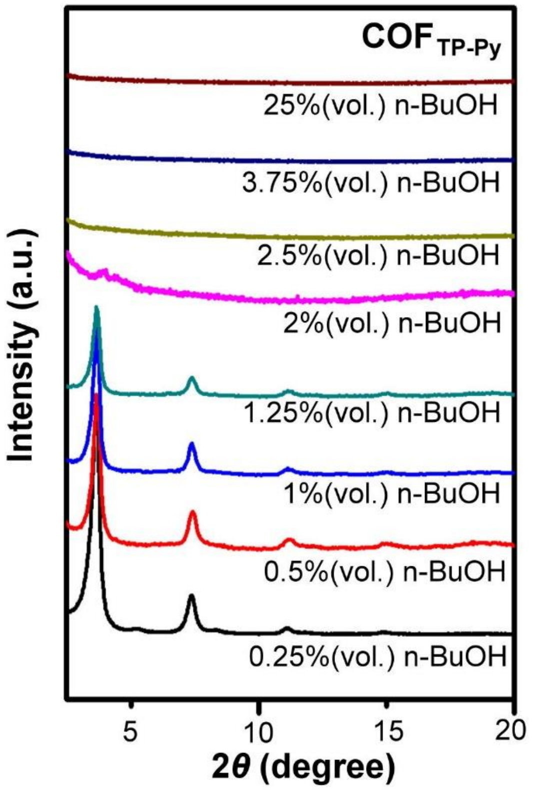

Supplementary Fig. 25. PXRD patterns of COF_{TP-Py}. The samples are grown for 1 hour in sc-CO_2 with 0.25% to 25% (vol.) n-BuOH.

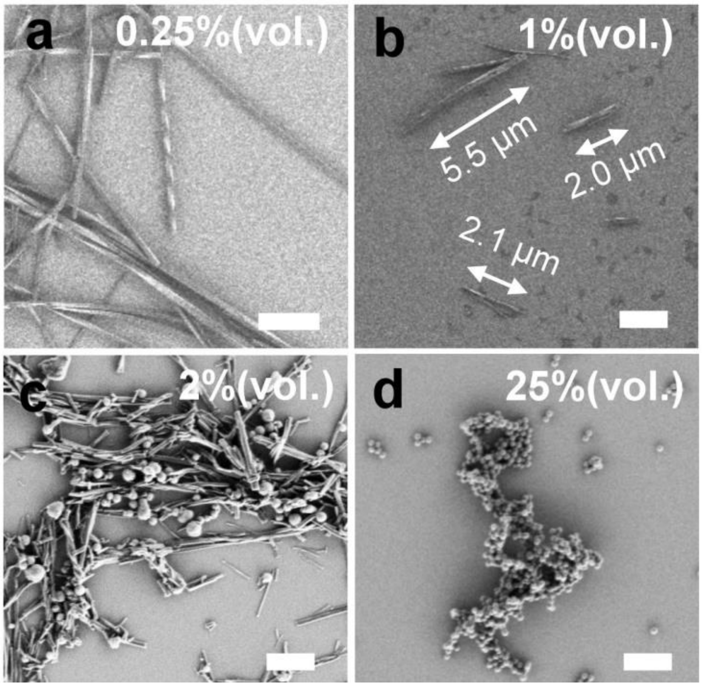

Supplementary Fig. 26. COF$_{TP-Py}$ grown in mixture of sc-CO_2 and n-BuOH. SEM images of COF$_{TP-Py}$ grown for 1 hour in sc-CO_2 with (**a**) 0.25% (vol.), (**b**) 1% (vol.), (**c**) 2% (vol.), (**d**) 25% (vol.) n-BuOH. The scale bars are 5 μm in **a**, 2 μm in **b-d**.

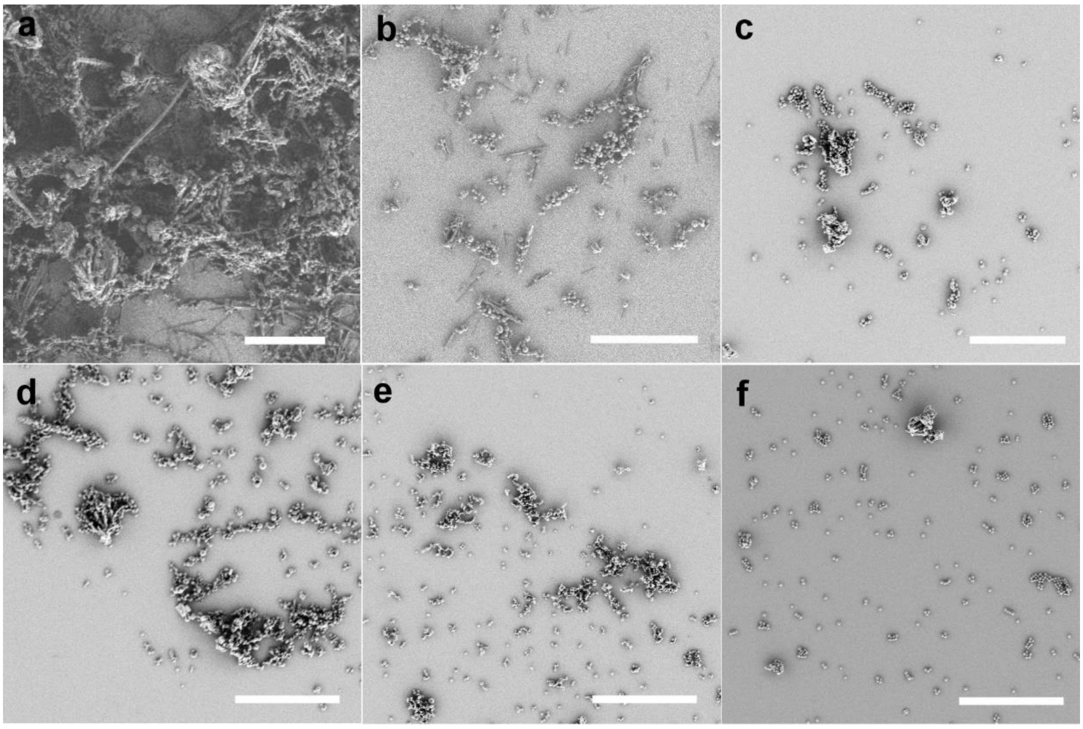

Supplementary Fig. 27. $COF_{TP\text{-}Py}$ grown in mixture of sc-CO_2 and n-BuOH. SEM images of $COF_{TP\text{-}Py}$ grown for 1 hour in sc-CO_2 with (**a**) 2% (vol.), (**b**) 2.5% (vol.), (**c**) 3.75% (vol.), (**d**) 6.25% (vol.), (**e**) 12.5% (vol.), (**f**) 25% (vol.) n-BuOH. The scale bars are 5 μm in **a**, 10 μm in **b-f**.

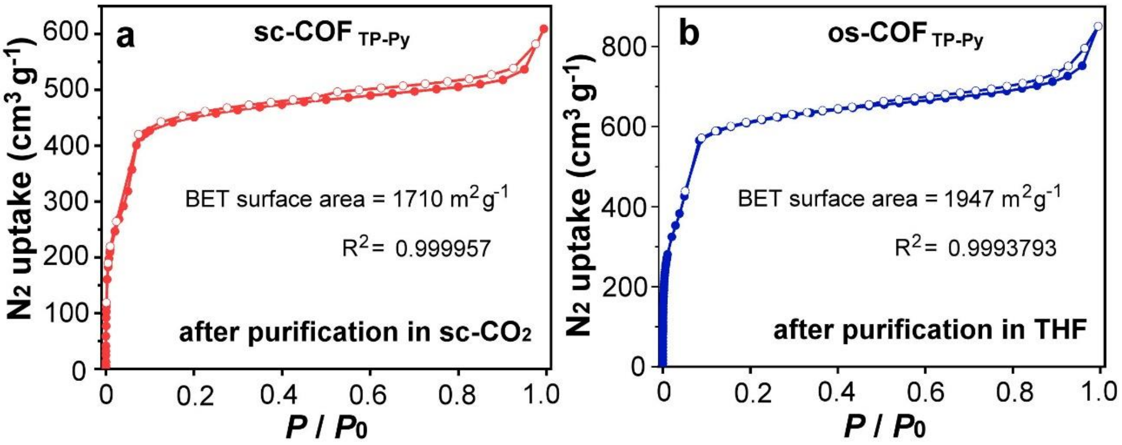

Supplementary Fig. 28. N_2 adsorption isotherms of the samples. a, sc-COF$_{TP-Py}$ after purification in sc-CO_2. **b,** os-COF$_{TP-Py}$ after purification in THF. This result clearly shows a type I isotherm. The sc-COF$_{TP-Py}$ purified in sc-CO_2 has a comparable surface area with os-COF$_{TP-Py}$ purified in THF, indicating the environmental friendliness of sc-COFs synthesis and treatment.

Description of Supplementary Videos

Supplementary Video 1.

The video collected by a cross-polarized optical microscope. It shows that the sc-$COF_{TP\text{-}Py}$ single crystals have uniform polarized light extinction over entire length, when the stage with samples is rotated from 0 ° to 180 °.

Supplementary Video 2.

The video collected by a cross-polarized optical microscope. It shows that the os-$COF_{TP\text{-}Py}$ doesn't have any polarized light extinction, when the stage with samples is rotated from 0 ° to 180 °.

Supplementary Reference

1. Wang, M. *et al.* Angle-dependent photoluminescence spectroscopy of solution-processed organic semiconducting nanobelts. *J. Phys. Chem. C* **121**, 12441−12446 (2017).

2. Yang, X., Lin, X., Zhao, Y., Zhao, Y. S. & Yan, D. Lanthanide metal-organic framework microrods: colored optical waveguides and chiral polarized emission. *Angew. Chem. Int. Ed.* **56**, 7853−7857 (2017).

3. Motamen, S. *et al.* Revealing order and disorder in films and single crystals of a thiophene-based oligomer by optical spectroscopy. *ACS Photonics* **3**, 2315–2323 (2016).

4. Huang, L., Tam-Chang, S. W., Seo, W. & Rove, K. Microfabrication of anisotropic organic materials via self-organization of an ionic perylenemonoimide. *Adv. Mater.* **19**, 4149–4152 (2007).

5. Che, Y., Yang, X., Balakrishnan, K., Zuo, J. & Zang, L. Highly polarized and self-waveguided emission from single-crystalline organic nanobelts. *Chem. Mater.* **21**, 2930–2934 (2009).

6. Yan, D., Jones, W., Fan, G., Wei, M. & Evans, D. G. Organic microbelt array based on hydrogen-bond architecture showing polarized fluorescence and two-photon emission. *J. Mater. Chem. C* **1**, 4138 (2013).

7. Narayan, T. C., Miyakai, T., Seki, S. & Dinca, M. High charge mobility in a tetrathiafulvalene-based microporous metal-organic framework. *J. Am. Chem. Soc.* **134**, 12932–12935 (2012).

8. Saeki, A., Ohsaki, S.-i., Seki, S. & Tagawa, S. Electrodeless determination of charge carrier mobility in poly(3-hexylthiophene) films incorporating perylenediimide as photoconductivity sensitizer and spectroscopic probe. *J. Phys. Chem. C* **112**, 16643–16650 (2008).

9. Wan, S. *et al.* Covalent organic frameworks with high charge carrier mobility. *Chem. Mater.* **23,** 4094–4097 (2011).
