# Supplementary material for: Ultra-fast single-crystal polymerization of large-sized covalent organic frameworks
Source: Nat Commun. 2021 Aug 23;12:5077. doi: 10.1038/s41467-021-24842-x (PMC8382702; doi:10.1038/s41467-021-24842-x)
Supplement: Supplementary file 3 — Description of Additional Supplementary Files [file 41467_2021_24842_MOESM3_ESM.pdf]

Description of Additional Supplementary Files

File Name: Supplementary Movie 1
Description: The video collected by a cross-polarized optical microscope. It shows that the sc-COFTP-Py single crystals have uniform polarized light extinction over entire length, when the stage with samples is rotated from 0° to 180°.

File Name: Supplementary Movie 2
Description: The video collected by a cross-polarized optical microscope. It shows that the os-COFTP-Py doesn’t have any polarized light extinction, when the stage with samples is rotated from 0° to 180°.
